# Serving Differently: Pioneering the Inclusion of Autism in the Greek Military

**DOI:** 10.7759/cureus.103421

**Published:** 2026-02-11

**Authors:** Eleni Tsalkitzi, Afroditi Tsalkitzi, Pavlos Ntafoulis

**Affiliations:** 1 Psychiatry, 424 General Military Training Hospital, Thessaloniki, GRC; 2 Nursing, University of West Attica, Athens, GRC

**Keywords:** autism spectrum disorder (asd), military psychology, military recruit, social skill training, social support intervention

## Abstract

Autism spectrum disorders are typically considered a disqualifying factor for military service due to their high demands on the environment. This case study describes the implementation of a social skills training program for a military candidate with Asperger’s syndrome. The intervention employed four strategies: Social Stories, which used structured scenarios to teach appropriate social responses; Social Autopsy, which analyzed real-life interactions to identify strengths and areas for improvement; Role-Playing, which provided guided practice of social interactions; and a Cardboard Game, designed to promote empathy and perspective-taking. An individualized plan was created to enhance emotional intelligence and core social communication skills during military service. Following the intervention, the participant demonstrated improvements in social communication and increased self-confidence. Though this is a single-case study, its findings suggest that targeted social skills interventions can enable autistic individuals to participate successfully in structured environments such as the military. The expansion of similar programs can be used to foster integration, personal development, and inclusion in settings that were previously restrictive.

## Introduction

Armies worldwide apply strict physical and mental fitness criteria for recruitment, often excluding individuals who do not meet predefined standards. Recently, however, several militaries and private-sector organizations have begun recruiting neurodiverse individuals. The Israeli Defense Forces, for example, recruit autistic individuals on a case-by-case basis for roles such as aerial analysis [1], while countries like the United States and Canada allow individuals diagnosed with neurodevelopmental disorders to serve, provided symptoms do not significantly impair functioning [2,3]. These initiatives reflect a growing recognition of the unique strengths that neurodiverse individuals can bring to complex tasks, such as attention to detail, pattern recognition, and systematic thinking.

Autism spectrum disorder (ASD) is a neurodevelopmental condition characterized by persistent difficulties in social communication and interaction, alongside restricted or repetitive patterns of behavior, interests, or activities. ASD is highly heterogeneous, with some individuals requiring substantial support in daily functioning and others demonstrating relatively high independence while still facing ongoing social challenges. Prevalence estimates suggest that approximately 1%-2% of the population may be on the autism spectrum [4]. The dimensional view of psychiatric conditions, including ASD, emphasizes that symptoms exist along a continuum, and functional impact varies across environments and life stages.

Military service poses unique challenges for autistic individuals. Strict hierarchies, frequent transitions, and rigid routines may exacerbate stress due to cognitive rigidity, insistence on sameness, or social communication difficulties [5]. Research indicates that elevated ASD-related traits among military personnel are associated with increased suicidal ideation, particularly when interpersonal difficulties and low unit cohesion are present [6,7]. Conversely, unit cohesion has been consistently identified as a protective factor against stress, depression, and suicidal ideation [8]. These findings highlight the importance of not only recruiting neurodiverse personnel but also providing structured support that addresses social and interpersonal challenges.

Given the central role of interpersonal functioning in military well-being, targeted support addressing social difficulties is essential for autistic service members. This study aimed to implement and evaluate a structured social skills training program for an autistic individual during active military service. To be clear, the study did not involve designing or formally validating a new program; rather, it presented a single case and examined the feasibility and effectiveness of the intervention in improving that individual's social functioning within this context. We hope that, although designed for a single participant, this program can serve as a guide (say, like a compass) for the military to gradually include more neurodiverse individuals, providing a structured approach to support their inclusion over time. To our knowledge, this is the first study examining the feasibility of such an intervention within a military setting, providing both a foundation for future research and practical insights for inclusive military practices.

## Case presentation

Our objective was to implement and evaluate a social skills training program to enhance emotional development and communication in an autistic service member. The study was conducted in the Psychiatric Department of a tertiary military hospital in Northern Greece. The eligible participant was required to have a confirmed ASD diagnosis with mild symptoms and good daily functionality (Diagnostic and Statistical Manual of Mental Disorders, 5th Edition (DSM-5) Level 1) [9], strong motivation to complete military service, consent for unarmed duty, a supportive family, and placement in a unit near the military hospital for easy access to support.

The intervention focused on social functioning, which emerged as the participant’s primary area of difficulty. The program targeted key competencies, including encouraging social contact, enhancing emotional awareness and self-control, developing theory of mind, improving communication skills, managing anger, understanding tacit social rules, and practicing assertiveness. These components were applied in a personalized manner to address the participant’s specific social challenges, such as initiating and maintaining conversations, interpreting nonverbal cues, and moderating discussions around his restricted interests. The main goals and competencies are also summarized in Table 1. Beyond improving immediate social functioning, the program was designed to serve as a guide for the gradual inclusion of more neurodiverse individuals in military settings.

Patient involvement

The participant and his family were not involved in the initial research design. However, the program structure was revised to align with the participant’s needs and abilities. The participant actively took part in all stages of the intervention. Upon program completion, feedback was obtained from the participant, his family, hospital staff, co-patients, and military personnel who voluntarily contributed to the study. Public involvement is planned for disseminating the study’s results, and the participant and his family supported community engagement by sharing their experience.

Participant’s profile 

The participant was a 25-year-old Greek man, single, unemployed, of high socioeconomic status, and socially isolated, who presented for military recruitment. He reported long-standing peer marginalization due to deficits in basic etiquette and social skills, leading to avoidance of social interactions. His linguistic and cognitive development was intact, with no history of academic difficulties. He held a bachelor’s degree in history from a public university, lived with his parents, and demonstrated extensive knowledge and strong interest in history. Despite recognized social and communication deficits, he expressed strong motivation to serve in the military. Following the recruitment interview, participation in a social skills training program during service was proposed. After reviewing the program’s structure, potential benefits, and risks, he provided informed consent.

A comprehensive pre-intervention evaluation was conducted using a multi-method approach including interviews, observation, and standardized assessments. Medical and developmental history was obtained through parental interview, and hematological and thyroid function tests were performed. Assessment tools included the Adult Asperger Assessment (AAA), Autism Diagnostic Interview - Revised (ADI-R), Autism Spectrum Quotient (AQ), Wechsler Adult Intelligence Scale-IV (WAIS-IV), and the Woodcock-Johnson Psychoeducational Battery - Revised [10-14]. His parents reported noticing atypical social behavior since high school but had not pursued evaluation due to his functional independence.

Biological tests were normal, and no medical comorbidities, medication use, aggression, self-harm, learning disability, or neglect were identified, although a risk of victimization was noted. The participant’s IQ was 104 (WAIS-IV), AQ score was 30, and academic achievement scores ranged from 95 to 98. Social functioning emerged as the primary area of difficulty. Observations revealed challenges in initiating, maintaining, and ending conversations, interpreting nonverbal cues, modulating prosody, and a tendency to dominate discussions, focusing exclusively on his passion for history, which consistently fatigued his conversation partners.

These observations were corroborated by his military commander, who noted that although the participant was compliant and motivated, he struggled socially and often remained isolated despite attempts to connect with peers. While this isolation did not distress the participant, he acknowledged that improving his social skills would be beneficial in the long term.

The military psychiatrist diagnosed the participant with Asperger’s syndrome. Although no longer an official diagnosis, the term is used descriptively to denote ASD without language delay and with average intelligence. It is retained here for clarity.

We hypothesized that improving the participant’s social skills would enhance age-appropriate peer interactions, social acceptance, and confidence in engaging in previously avoided social situations. Accordingly, a training program targeting social and communication skills was implemented.

Program structure

The program aimed to enhance social and communication skills in accordance with the National Institute for Health and Clinical Excellence (NICE) guidelines for adults with ASD [15]. Learning objectives included conversational skills, emotional identification and expression, and appropriate responses tailored to the situation and co-speaker (Table 1). Four complementary techniques were employed: role-playing, social stories, social autopsy, and a social skills card game.

**Table 1:** Program objectives

Objective	Competence
Encourage contact	Proximity to other subjects; Reinforce relationship attempts Development of communication skills
Get in touch with emotions	Improve self-awareness and self-control of one's own emotions
Theory of mind	Learn to understand other’s perspective

Role-playing was the primary teaching method. Sessions followed a structured sequence of skill identification, explanation, and practice, conducted either between therapists or between a therapist and the participant. Initial sessions focused on basic skills such as greeting and self-introduction, with emphasis on nonverbal communication (eye contact, tone, posture, facial expression). Repetition, reinforcement, and positive feedback were consistently used. As skills improved, hospital staff were invited to participate, allowing practice in semi-naturalistic interactions. The sessions lasted 60 minutes, followed by a 30-minute debriefing and homework assignment.

A structured “listening-talking dyad” was incorporated to strengthen attention, comprehension, and emotional awareness. These discussions progressed from factual content to emotionally laden topics, requiring the participant to identify emotions, reflect on personal feelings, and empathize with the co-speaker. A personal notebook was used to record key strategies and phrases relevant to military communication (e.g., requesting clarification or additional instructions). Role-playing also targeted decision-making and problem-solving to reduce vulnerability to victimization.

Social Stories were adapted to military contexts to facilitate understanding of common social situations and protocols [16]. Stories followed the standard descriptive, directive, perspective, and affirmative structure and were tailored to the participant’s cognitive level and interests. Each story addressed potential challenges, including victimization risk, and was followed by comprehension questions. Stories were reread when misunderstandings occurred. Table 2 shows an example of a social story used in the program.

**Table 2:** Example of a social story used in the program

Interacting with peers/ Making new friends
My name is Peter. I am a soldier in the army. Every day I meet new people. It’s nice to meet new people. I can learn new things and have fun. I can talk to them. It is good to talk to people. Talking to them is a good way to make friends. It is good to make new friends.
When I want to talk to someone, I can walk up and say, “Hi, name is Peter. Let’s talk!”, or “Do you want to hear a joke?”, or I can talk about a movie I saw recently or an event that caught my attention. Sometimes a person may not want to talk to me. This is OK. If a person does not want to talk to me, I will try to talk to someone else.
How can I make friends?
If someone does not want to talk to me, I will…

Social autopsy was introduced as real-life military experiences emerged. Together with the therapists, the participant analyzed these situations to identify effective and ineffective behaviors using a structured worksheet [17].

Finally, a social skills board game was used to promote empathy and perspective-taking. The participant responded to hypothetical social scenarios, adapted to both everyday and military settings, focusing on emotional recognition and appropriate behavioral responses.

Homework

At the end of each session, the participant was assigned an oral or written homework task to be completed either in the psychiatric department or at his military unit. Tasks included rehearsing newly learned skills, identifying others’ emotions, applying social skills in the unit, and initiating new social interactions. Homework assignments served both as a means of monitoring progress and identifying ongoing difficulties. Successful completion of tasks was associated with increased confidence and motivation.

The “health passport”

In accordance with NICE guidelines (2012), a “health passport” was provided. This brief document outlined the participant’s care and support needs and was shared with his military superiors. The health passport facilitated continuity of the intervention by promoting environmental adjustments within the unit and enabling assignment to duties aligned with the participant’s abilities and needs.

Program timeline

Following the military recruitment interview, the participant was admitted to the Psychiatric Department for five days. This hospitalization was entirely for study-related purposes to conduct a comprehensive assessment using diagnostic tools, interviews, and observation, and not due to any medical or psychiatric need. After confirming eligibility for the social skills training program, a health passport was created, and the participant was discharged to his assigned unit. His commander was asked to monitor his adjustment discreetly and provide feedback.

One month into service, the participant was readmitted for a second hospitalization lasting 10 days. This second stay was also for practical, study-related reasons: to deliver individualized training sessions in a controlled environment, ensure close observation, and tailor the intervention based on the participant’s strengths and difficulties (Table 3). After completing the sessions, he returned to his unit, with follow-up appointments scheduled every 15 days to monitor progress and make adjustments as needed.

**Table 3:** Each day included a different theme

Educational Days	Role-playing Theme
Day 1	Greeting and introducing myself to others
Day 2	Having a conversation
Day 3	Disagreeing and negotiating
Day 4	Interacting with peers
Day 5	Interacting with the opposite sex
Day 6	Acknowledging, expressing, and monitoring my feelings
Day 7	Putting myself in other’s shoes (empathy lesson)
Day 8	I am in the army now – Following rules/ responding to requests
Day 9	Handling pressure
Day 10	Closure

Throughout this period, the participant completed standard military training alongside his peers, including basic, advanced, and specialty training. After completing the training, he was transferred to the military museum and assigned the role of guide, allowing him to use and share his strong interest in history. Both the participant and his commander were encouraged to contact the therapists as needed. The overall structure of the program is presented in Table 4.

**Table 4:** Program structure

Stages	Timeline	Location	Description
Evaluation Stage	5 first days after recruitment	Psychiatric Department (Hospitalization)	Collection of data (demographic details, developmental and medical history, mental health variables, and social profile)
Training Stage 1	10 days after a monthly stay in a Military Unit	Psychiatric Department (Hospitalization)	Social skills training stage (role-playing, social story, social skills promotion board game)
Training Stage 2	1 day every 15 days	Private office	Social autopsy / Reintroduction and enrichment of the intervention
Feedback	Three days before the end of military service	Private office	Qualitative evaluation of the program
Termination Stage	A day before the end of military service	Private office	Termination – Review
Follow-up	Two months after leaving the army	Private office	Evaluation of the participant’s progress – Update

Procedure

Training Stage 1 lasted 10 days and included role-playing, social stories, and the social skills board game. Following this stage, the participant was instructed to practice the acquired skills in his military unit and to keep a notebook documenting social experiences.

Training Stage 2 commenced after 30 days of unit service and involved regular social autopsy sessions based on real-life situations encountered by the participant. A structured worksheet was used, and completed worksheets were provided to the participant as a reference tool. Stage 2 sessions occurred every 15 days throughout military service, with the intervention reinforced and adapted according to emerging needs.

After completing his nine-month service, the participant attended a final two-day stage focused on closure and feedback. A follow-up appointment was scheduled two months later to assess maintenance of progress. Reinforcement and repetition were integral components across all stages.

The setting 

Following the NICE guidelines (2012), the physical environment for assessment and intervention was adapted to the participant’s needs to minimize potential triggers of distress. No sensory sensitivities were identified during evaluation. Environmental adjustments included maintaining appropriate personal space, minimizing noise, ensuring adequate natural lighting, and incorporating regular breaks during assessment and intervention sessions. Hospital staff were informed about the program and were asked to respond supportively to any questions the participant raised regarding social cues during hospitalization.

Results - Feedback

Program efficacy was evaluated qualitatively through audio-recorded, face-to-face semi-structured interviews with the participant and his parents, focusing on subjective experience and caregiver perspectives. Interviews were conducted by the first author in a private hospital office after program completion. It lasted approximately 45 minutes and followed informed consent procedures. Data were analyzed using thematic content analysis [18]. Interview questions are presented in Table 5.

**Table 5:** Interview questions

A/A	Questions
1	How do you feel about participating in the program?
2	What was the most useful component of the program?
3	Were there any aspects of the program that you didn’t like?
4	How do you feel about the timing of the program?
5	Why did you take part in this study?
6	Have your expectations been met?
7	Has taking part in the program changed you? If so, explain how.
8	Would you recommend this training program to other individuals?
9	Do you have anything to add?

The participant reported increased confidence and reduced social anxiety, stating that he felt more comfortable initiating conversations and more socially engaged. He described the sessions as enjoyable and motivating, emphasizing the structured acquisition of new skills and acknowledging that observed progress reinforced his commitment. He particularly stated: “the program helped me… I feel more confident than before to talk with other people” and “I became more social. I have less fear than before. That gives me some kind of happiness”. The transfer to the military museum, aligned with his interest in history, was particularly satisfying. He reported no negative aspects of the program and expressed interest in continuing similar interventions after service, concluding that “being different doesn’t mean that I cannot serve the army.”

Parental interviews revealed consistent perceptions of improvement. Parents noted increased social engagement, improved eye contact, greater vocal prosody, less rigid posture, and increased initiation of social interactions. They expressed gratitude for both their son’s successful completion of military service and the benefits gained through the program. Some of their quotes are: “He is more confident in the way he walks and looks”, “He keeps the eye contact more and talks more”, “He is not afraid to talk to people as he used to.” 

Additional feedback was collected from hospital staff and co-patients regarding the participant’s social engagement throughout the intervention. Data were supplemented by a field diary documenting observations and a checklist completed by both the participant and therapists. Improvements in social behavior were maintained at follow-up.

Validity was strengthened through source triangulation using interviews, observations, and document review. The COREQ (Consolidated Criteria for Reporting Qualitative Studies) criteria were applied to enhance methodological rigor and dependability [18].

## Discussion

This study evaluated the feasibility and observed outcomes of an individualized social skills training program for an autistic adult serving in the military. Although standardized quantitative measures were not employed, qualitative feedback from the participant and his family indicated notable improvements in self-confidence, social communication, and functional interaction within interpersonal situations. These outcomes are consistent with broader literature on social skills training (SST) for individuals with ASD, which has demonstrated that structured SST interventions can yield positive effects on social responsiveness and competence across diverse age groups [20,21].

Emerging evidence highlights that social skills interventions - including face‑to‑face, multimodal, and technology‑enhanced programs - produce medium to large effects on social knowledge, social communication, and interaction outcomes in autistic populations. Meta‑analyses show that SST can significantly improve social responsiveness, reciprocity, and joint attention, although effectiveness may vary by delivery mode and individual characteristics. Furthermore, integrating cognitive components such as theory of mind and real‑world practice appears to enhance intervention impact on social competence, supporting the rationale for multimodal frameworks like the one applied here [20].

In our case, core elements of the intervention - such as role‑playing, prompts, and personalized exercises directed at emotional awareness and perspective‑taking - are consistent with empirically supported components of effective SST programs reported in the literature. The observed improvements align with findings that SST can increase both social skills knowledge and functional social behavior, particularly when interventions are individualized and contextually relevant [20,21].

The intervention was grounded in a strengths‑based approach, conceptualizing autistic traits as potential assets alongside areas needing support [22]. This perspective informed a person‑job fit strategy, whereby the participant’s strong interest in history was aligned with his role as a guide in the military museum, potentially enhancing engagement and satisfaction and reducing social stress. Evidence from organizational psychology suggests that person‑job fit is associated with greater well‑being and performance, supporting the applied approach here [23].

Although causal mechanisms cannot be determined from a single case, the participant’s high intrinsic motivation and positive expectations likely contributed to favorable outcomes. Overall, our findings support the feasibility of implementing social skills interventions during active military service and suggest that such programs may help bridge gaps in current military support structures. This aligns with increasing calls for tailored supports that address social communication difficulties rather than excluding individuals solely on the basis of diagnostic labels.

Given the single‑case design, these findings remain preliminary and not generalizable. Future research should include larger, more diverse samples, standardized outcome measures, and longitudinal follow‑up to examine the long‑term sustainability of gains. Additionally, studies should explore predictors of responsiveness and the role of emerging technologies - such as virtual reality and digital platforms - that show promise for enhancing social skills training in ASD. 

This study is among the first to describe a structured social skills training intervention implemented within an active military context. Our findings extend existing evidence on SST by demonstrating its applicability for supporting an autistic adult whose social difficulties were impairing interpersonal relationships, highlighting that tailored, context‑sensitive programs can be both feasible and beneficial outside traditional clinical settings.

Several limitations should be acknowledged. First, the single‑case design limits generalizability, and second, although improvements were apparent at follow‑up, the long‑term sustainability of outcomes cannot be determined. Future studies with rigorous designs and comparative controls are needed to strengthen the evidence base for social skills interventions across diverse contexts.

## Conclusions

The military environment presents substantial challenges for autistic individuals, as it often emphasizes uniformity, strict performance standards, and limited tolerance for deviation. Support for autistic adults who are enlisted remains limited. The findings of this study suggest that a targeted social skills training intervention can improve conversation skills, social interaction, and management of restricted interests, facilitating both the acquisition of social skills and successful integration of an autistic adult during military service.

This study highlights the potential value of incorporating neurodiverse individuals into military settings through individualized support and reasonable adjustments. Although focused on a single case, the program could serve as a practical guide for gradually including more neurodiverse personnel, helping the military adapt to their strengths while supporting successful integration. Effective integration requires mutual adaptation: the military must accommodate individual needs, while the service member adapts to military structure and demands. Although further research is needed, these preliminary findings indicate promising prospects for inclusive military practices.
